# Intramuscular coherence during challenging walking in incomplete spinal cord injury: Reduced high-frequency coherence reflects impaired supra-spinal control

**DOI:** 10.3389/fnhum.2022.927704

**Published:** 2022-08-03

**Authors:** Freschta Zipser-Mohammadzada, Bernard A. Conway, David M. Halliday, Carl Moritz Zipser, Chris A. Easthope, Armin Curt, Martin Schubert

**Affiliations:** ^1^Spinal Cord Injury Center, Department of Neurophysiology, Balgrist University Hospital, Zurich, Switzerland; ^2^Department of Biomedical Engineering, University of Strathclyde, Glasgow, United Kingdom; ^3^Department of Electronic Engineering, University of York, York, United Kingdom; ^4^York Biomedical Research Institute, University of York, York, United Kingdom; ^5^Cereneo Foundation, Center for Interdisciplinary Research, Vitznau, Switzerland

**Keywords:** spinal cord injury, EMG-EMG coherence, intramuscular coherence, gait, visually guided walking, motor control, balance control, center of mass

## Abstract

Individuals regaining reliable day-to-day walking function after incomplete spinal cord injury (iSCI) report persisting unsteadiness when confronted with walking challenges. However, quantifiable measures of walking capacity lack the sensitivity to reveal underlying impairments of supra-spinal locomotor control. This study investigates the relationship between intramuscular coherence and corticospinal dynamic balance control during a visually guided Target walking treadmill task. In thirteen individuals with iSCI and 24 controls, intramuscular coherence and cumulant densities were estimated from pairs of Tibialis anterior surface EMG recordings during normal treadmill walking and a Target walking task. The approximate center of mass was calculated from pelvis markers. Spearman rank correlations were performed to evaluate the relationship between intramuscular coherence, clinical parameters, and center of mass parameters. In controls, we found that the Target walking task results in increased high-frequency (21–44 Hz) intramuscular coherence, which negatively related to changes in the center of mass movement, whereas this modulation was largely reduced in individuals with iSCI. The impaired modulation of high-frequency intramuscular coherence during the Target walking task correlated with neurophysiological and functional readouts, such as motor-evoked potential amplitude and outdoor mobility score, as well as center of mass trajectory length. The Target walking effect, the difference between Target and Normal walking intramuscular coherence, was significantly higher in controls than in individuals with iSCI [*F*(1.0,35.0) = 13.042, *p* < 0.001]. Intramuscular coherence obtained during challenging walking in individuals with iSCI may provide information on corticospinal gait control. The relationships between biomechanics, clinical scores, and neurophysiology suggest that intramuscular coherence assessed during challenging tasks may be meaningful for understanding impaired supra-spinal control in individuals with iSCI.

## Introduction

Human walking involves complex stereotyped motion sequences that are continuously adjusted to environmental demands that challenge progression. The integration of multimodal sensory inputs and supra-spinal commands, including visuomotor control, are required to achieve effective adaptive and controlled stepping (Rossignol et al., 2006; Matthis et al., 2017; Pearcey and Zehr, 2019). The supra-spinal and multimodal sensory command processes fail to be properly conducted to and between spinal cord levels following spinal cord injury or degeneration affecting spinal cord tracts. Yet while many individuals with incomplete spinal cord injury (iSCI) can regain walking ability, limb- and balance coordination are impaired (Barbeau et al., 2006; Awai and Curt, 2014; Easthope et al., 2018; Malik et al., 2019), resulting in reduced capacity to adapt to challenging walking when precision in foot placement is a requirement (e.g., walking over irregular surfaces) (Leroux et al., 1999; Desrosiers et al., 2014; Mohammadzada et al., 2022). Whilst such functional deficits are a consequence of spinal tract damage, the integrity of supra-spinal control of human gait is difficult to assess, and the neural mechanisms of visuomotor coordination during gait in individuals with iSCI remain poorly understood.

During outdoor walking on uneven terrain, continuous visuomotor and proprioceptive integration is needed for reactive and anticipatory gait adjustments (Marigold et al., 2011). Individuals with iSCI may utilize different adaptation strategies compared to controls, as shown in studies investigating individuals with iSCI during obstructed walking (Leroux et al., 1999; Ladouceur et al., 2003) and this is likely to be attributed to impaired transmission of descending neural drive to the muscles, which can be assessed non-invasively with coherence analysis (Halliday et al., 1995). Coherence measures provide an estimate of a coupled relationship between two simultaneously recorded signals in terms of common coherent components (Plankar et al., 2013). Corticomuscular coherence represents a coupling feature between muscle activity and cortical activity (Conway et al., 1995), while intermuscular coherence quantifies the coupling or common drive between two separate muscles. Intramuscular coherence quantifies the shared common drive occurring within the active motor units of the same muscle. Coherence analysis can serve as a reliable method to explore sensorimotor integration non-invasively by observing changes in specific physiologically relevant frequency bands. While intra- and intermuscular coherence in the alpha-band (8–12 Hz) is debated to have its origin in spinal- and subcortical systems, as shown in studies analyzing intermuscular coherence in lower (Norton et al., 2003) and upper limbs (Boonstra et al., 2009); the high-frequency bands, such as beta- (15–32) and gamma-band (35–60 Hz), are associated with activation from supra-spinal systems and provide insight on descending influences to muscles during movements as shown in corticomuscular coherence studies investigating sensorimotor cortex contributions to the control of upper limb and in lower limb muscles during walking in neurological intact adults (Conway et al., 1995; Salenius et al., 1997; Halliday et al., 1998; Baker, 2007; Petersen et al., 2012).

In individuals with iSCI and stroke, intra- and intermuscular coherence between muscle pairs in the high-frequency bands are strongly reduced during regular treadmill walking compared to controls (Hansen et al., 2005; Nielsen et al., 2008; Barthélemy et al., 2010); similarly, in upper motor neuron disease, intermuscular coherence in the high-frequency band is either absent or reduced (Fisher et al., 2012). These findings suggest that an intact supra-spinal drive is needed for normal function. Accordingly, high-frequency coherence between and within co-active muscles may be an indicator for supra-spinal drive, notwithstanding that synchronizing contributions from other neural circuits not characterized by common frequency components in EMG records will not be observable (e.g., extrapyramidal motor system, reticulospinal, and ascending sensory tracts).

Precision motor tasks that demand and engage attention may increase coherence in the high-frequency band as shown in corticomuscular coherence studies of the sensorimotor cortex and the contralateral hand (Kristeva-Feige et al., 2002; Kristeva et al., 2007), possibly due to enhanced motor cortex task related contributions to the shaping of motor output (Drew et al., 1996, 2008), and planning of movements (Spedden et al., 2022). In the lower limb, an example of this is observed during visually guided walking where increased Tibialis anterior intramuscular and corticomuscular beta- and gamma-band coherence is observed during the swing phase when compared to normal walking in a control cohort (Jensen et al., 2018; Spedden et al., 2019a). Thus, using a visually guided walking paradigm provides an opportunity to investigate the relationship between high-frequency coherence and adaptive locomotor capacity. By investigating a cohort of individuals with iSCI, we strive to understand to what extent spinal cord damage impacts high-frequency coherence and how this relates to the capacity for continuous locomotor adaptation in iSCI.

Therefore, our primary aim was to test the hypothesis that, compared to controls, modulation of high-frequency intramuscular coherence is reduced or lacking in individuals with iSCI when challenged with a visually guided Target walking task (TW). We further explored how high-frequency intramuscular coherence relates to kinematics and impairment of gait adaptation in individuals with iSCI to assess whether coherence measures can serve as a potential marker for the pathophysiological underpinnings of impaired gait adaptation.

## Materials and methods

### Participants

For this study, twenty-four controls without a history of neurological disease and thirteen individuals with iSCI from Balgrist University Hospital were recruited and provided written informed consent. Individuals were older than 18 years old, could stand without physical assistance for over 2 min, and were either in the subacute (3–6 months post-injury) or chronic (≥12 months post-injury) stage. All individuals with iSCI were required to have partially preserved or reacquired walking ability. Individuals were also included if they were dependent on walking aids for recreational activities (see Table 1). Individuals were not included when they had a neurological impairment other than iSCI or cognitive impairments liable to interfere with task performance. Both subacute and chronic individuals with iSCI were included in order to cover a broad range of walking abilities. The study was approved by the Zurich cantonal ethics Committee (BASEC-Nr. 2017-01780). All experiments were conducted in accordance with the current 2013 revision of the Declaration of Helsinki.

**TABLE 1:** Incomplete spinal cord injury demographics.

ID	Age (y)	Height (cm)	Sex (m/f)	Walking speed (m/s)		Mean step length (m)		Mean step width (m)		ML accuracy (cm)	AP accuracy (cm)	AIS	Level of injury	Time since injury (y)	Type of Injury	Total sensory score (right)	LEMS score		Max MEP (right TA)	Mean SCIM (max 8)	Walking aids
				NW	TW	NW	TW	NW	TW								R	L			
01	79	170	m	0.35	0.3	0.24	0.35	0.17	0.16	–1.5	4	D	L3	24	Traumatic	50	20	22	0.23	na	Yes
02	42	173	m	0.4	0.4	0.33	0.34	0.13	0.16	na	na	D	C3	1	Degenerative	90	25	25	0.9	5	No
03	65	175	m	1.2	1	0.58	0.45	0.09	0.18	–0.2	7.7	D	C4	1	Traumatic	112	25	25	0.43	6	No
04	57	176	m	0.8	0.8	0.52	0.39	0.09	0.16	–1.7	6.7	D	T4	0.3	Traumatic	71	25	25	0.39	7	No
06	36	180	m	0.8	0.8	0.53	0.43	0.17	0.19	–0.27	6	D	T7	5	Traumatic	112	25	25	0.05	5	No
07	69	167	f	0.6	0.6	0.36	0.40	0.19	0.2	–0.5	6.1	D	C5	6	Traumatic	102	23	23	0.09	3	No
09	48	182	m	0.9	0.8	0.58	0.40	0.15	0.17	–3	11.5	D	L3	18	Traumatic	100	24	24	0.94	7	No
10	58	163	f	0.8	0.6	0.54	0.41	0.14	0.18	–0.7	7.9	D	T4	2	Toxic	80	25	25	0.13	1	Yes
11	58	170	f	1	1	0.61	0.4	0.11	0.16	–1.3	9	D	T7	11	Degenerative	112	25	25	0.63	8	No
13	70	170	m	0.8	0.7	0.52	0.41	0.15	0.2	–2.4	7.6	D	T4	7	Tumor	112	25	25	0.38	4	No
14	66	170	m	0.8	0.7	0.44	0.39	0.15	0.16	–0.9	5.5	D	T3	14	Tumor	76	25	25	0.12	8	No
15	67	177	m	0.55	0.6	0.55	0.43	0.09	0.17	–1.7	1	D	L1	8	Tumor	94	19	16	0.31	6	Yes
16	54	184	m	0.7	0.6	0.55	0.4	0.14	0.16	–0.8	5.4	D	T12	6	Traumatic	90	25	25	0.2	5	No
Median	58	173		0.8	0.7	0.53	0.4	0.14	0.17	–1.1	6.4			6		94	25	25	0.31	5.5	
IQR	13	7.8		0.2	0.2	0.14	0.03	0.05	0.02	1.1	2.4			10		33	1.25	1.25	0.35	2.5	
Stats				*p* = 0.02		*p* = 0.006		*p* < 0.001													

y, years; m, male; f, female; NW, Normal walking; TW, Target walking; ML, medio-lateral; AP, anterio-posterior; AIS, American Spinal Injury Association (ASIA) impairment scale; LEMS, Lower extremities motor score; R, right; L, left; MEP, motor evoked potential; SCIM, spinal cord independence measure; na, not available; IQR, interquartile range; Stats, Statistics; paired wilcoxon test.

### Recordings

Surface EMGs were recorded wirelessly from proximal (TAp) and distal (TAd) sites of the right Tibialis anterior muscle. Recording sites were prepared by carefully shaving, abrading, and disinfecting the skin areas of interest before attaching bipolar EMG adhesive hydrogel electrodes (Kendall, Covidien) and wireless EMG sensors (myon AG, Switzerland, 2 kHz sampling rate, 10 – 500 Hz bandpass filter).

Full-body kinematics were recorded using a passive infra-red motion capture system (Vero, Vicon Motion Systems Ltd, Oxford, United Kingdom) operating at 100 Hz and processed in Nexus 2.2.3 (Vicon, Oxford, United Kingdom) and with custom-written MATLAB scripts (MATLAB R2017b) using 42 reflective markers placed on bony landmarks (diameter = 14 mm). For the step cycle identification, markers were placed on the heel and the second toe (metatarsal 2). The times of heel strike (HS) and toe off were calculated from the zero-crossing of heel and toe marker velocity (Zeni et al., 2008). Approximated center of mass (CoM) was defined as the midpoint between left posterior spina iliac to right anterior spina iliac and left anterior spina iliac to right posterior spina iliac (Havens et al., 2018; Bannwart et al., 2020).

### Coherence analysis

EMG signals were processed with custom-written MATLAB scripts. Recordings were zero-phase bandpass filtered (10–500 Hz), full-wave rectified, and normalized to unit variance before analysis in the frequency and time domain. Full-wave rectification suppresses any information related to the motor unit action potential shape (Farmer and Halliday, 2014).

Intramuscular coherences were investigated between TAp and TAd during the entire gait cycle and analyzed with the Neurospec routines using Type 1 analysis (Halliday et al., 1995) (Neurospec 2.0^1^). A complete gait cycle was defined as one stride going from one HS to the next HS with the same leg. The discrete Fourier transform (DFT) was constructed from each non-overlapping stride with a DFT segment length of 4096 samples (4096 samples/2000 Hz = 2048 ms), with zero padding used for strides with fewer samples, resulting in a frequency resolution of 0.49 Hz. Coherence measures assess the linear association between two rectified EMG signals on a scale from 0 to 1 in the frequency domain. The coherence function is defined at frequency λ as


(1) $${\left|{\text{R}}_{\mathrm{xy}}\left(\lambda \right)\right|}^{2}=\frac{{\left|{\text{f}}_{\mathrm{xy}}\left(\lambda \right)\right|}^{2}}{{\text{f}}_{\mathrm{xx}}\left(\lambda \right){\text{f}}_{\mathrm{yy}}\left(\lambda \right)}$$

*f*_*xy*_ (λ) denotes the cross-spectrum between EMG signals *x* and *y*. It is normalized by dividing it by the power spectrum of one EMG signal *f*_*xx*_(λ) multiplied by that of the other EMG signal *f*_*yy*_(λ). Estimates of the cumulant density function characterize the covariance of the signals in the time domain and give information related to the timing of shared inputs to the motor units contributing to the EMG recordings. It is defined as the inverse Fourier transform of the cross-spectrum and is a function of time lag *u*


(2) $${\text{q}}_{\mathrm{xy}}\left(u\right)=\int_{-\pi }^{\pi }{\text{f}}_{\mathrm{xy}}\left(\lambda \right){\text{e}}^{i\lambda u}d\lambda$$

A central peak around zero lag reflects the synchronous activity of populations of motor units and suggests the presence of common synaptic input to the motoneurons within the activated motor pool (Halliday et al., 1995, 2003).

### Pooled coherence and cumulant density estimates

Pooled coherence and pooled cumulant density measures (Amjad et al., 1997) are helpful to assess systematic task-dependent modulations in the correlation pattern in the gait cycle across subjects (Halliday et al., 2003). Pooled estimates, thus, allow for inferences at the population level in each group, combining all data from each group in a single representative estimate. This study uses the modulation pattern of pooled coherence to determine the frequency bands of interest for further in-depth single-case analysis.

### Clinical assessment

A neurological examination following International standards for neurological classification of spinal cord injury (Kirshblum et al., 2011) was performed at enrollment in individuals with iSCI. Motor-evoked potentials (MEPs) of the Tibialis anterior muscle were acquired from individuals in a supine position using single-pulse transcranial magnetic stimulation (TMS; Magstim-200, Magstim Company, Carmarthenshire, Wales, United Kingdom). A double cone coil was used to achieve focal stimulation of the vertex, and MEPs were recorded from the Tibialis anterior muscle with disposable surface EMG electrodes. The individuals were asked to relax and not voluntarily contract the Tibialis anterior muscle. Maximum stimulus output was set to evoke reproducible MEPs at maximum amplitude. In compliance with ethical guidelines, MEPs were not assessed in control participants to avoid unwarranted experimental measurements on volunteers.

### Experimental set-up

Participants walked at a self-selected walking speed with their typical, comfortable footwear on a split-belt treadmill with two integrated, synchronized force plates (GRAIL, Gait Realtime Analysis Interactive Lab, Motek Medical B.V., Netherlands). The participants were secured with a harness and were allowed to hold the handrails if needed. The participants completed the TW and Normal walking (NW) tasks in random order.

### Target walking

Target walking is a 3 min visually guided walking task requiring the participants to precisely step on to moving circular targets projected onto the treadmill belts. This task is in detailed explained and investigated previously (Mohammadzada et al., 2022) and summarized below.

**FIGURE 1:**
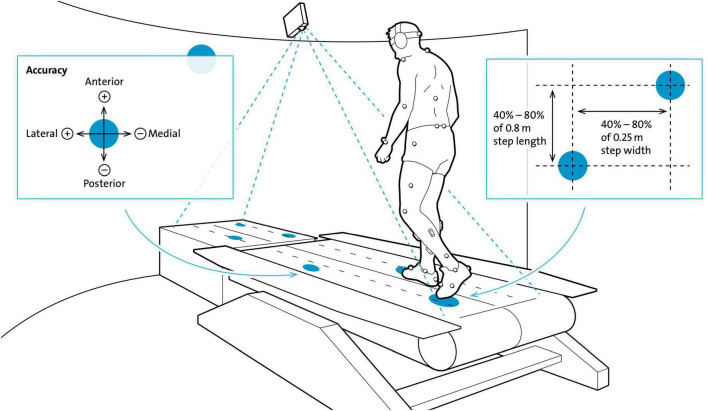
Experimental set-up for the Target walking task. Participants walked on moving white circular targets (diameter = 10 cm, here blue) projected on the black treadmill (here white) with their preferred walking speed. Circular targets moved at the same speed and thus, gave the impression of being stationary. Step length and width were changed based on a variability of 40–80% of 0.8 m and 40–80% of 0.25 m, respectively. Participants had to precisely step on to the center of the targets which was tracked by a reflective marker placed on the second toe. Accuracy of foot placement was recorded in the anterior-posterior (AP) and medio-lateral (ML) directions. The harness that was worn for safety and the handrails are not shown in this schematic figure of the Motek GRAIL treadmill system. Modified from Mohammadzada et al. (2022).

The circular targets (10 cm diameter, white dots) were projected onto and in front of the right and left treadmill belt (Figure 1) and moved toward the participant at the speed of the treadmill belt. Thus, the targets evoked the impression of being stationary compared to the moving treadmill surface (Motek Forcelink, Amsterdam, Netherlands, version 3.34.1). The participants were able to anticipate upcoming targets over three to four step cycles, since the targets were presented with a lead-in of greater than two stride lengths (Matthis et al., 2017). Adjustments of step cycle length (randomly distributed between 40 and 80% of 0.8 m step length) and width (randomly distributed between 40% and 80% of 0.25 m step width) were required to successfully step on to the targets.

In addition to standard markers for gait analysis, foot placement was tracked by a 14 mm reflective marker placed on the base joint of the second toe – metatarsal 2 (MT2). Placement precision was calculated by the distance (error) from MT2 to the center of the target in anterior-posterior (AP) and medio-lateral (ML) directions. Stepping medially and posteriorly from the center of the target resulted in negative error values, whereas stepping laterally and anteriorly resulted in positive error values. Errors were calculated once per step at midstance and recorded for offline analysis; participants did not receive feedback on errors. Due to technical reasons, accuracy measures in the ML- and AP-direction were introduced from participant 15 onward (see Table 2). Before data collection commenced, participants were provided with a 1 min task familiarization period.

**TABLE 2:** Participant demographics.

ID	Age (years)	Height (cm)	Sex (m/f)	Walking speed (m/s)		Mean step length (m)		Mean step width (m)		ML accuracy (cm)	AP accuracy (cm)
				NW	TW	NW	TW	NW	TW		
05	30	188	m	0.95	0.95	0.6	0.51	0.1	0.3	na	na
06	26	173	m	0.9	0.9	0.6	0.41	0.1	0.2	na	na
08	36	172	m	1.32	1.2	0.7	0.56	0.12	0.2	na	na
09	31	169	f	1.01	1	0.57	0.41	0.15	0.2	na	na
10	27	173	f	1.45	1	0.7	0.4	0.1	0.2	na	na
11	48	175	f	1	0.8	0.59	0.36	0.08	0.16	na	na
12	25	185	m	1.2	1.1	0.6	0.45	0.1	0.2	na	na
13	28	159	f	0.9	0.9	0.5	0.4	0.08	0.18	na	na
15	25	160	f	1.2	1.1	0.64	0.4	0.1	0.2	0.01	8.4
16	31	167	f	0.9	0.8	0.5	0.41	0.15	0.2	na	na
17	29	166	f	1.1	1.1	0.6	0.4	0.12	0.16	na	na
18	26	171	f	1.2	1	0.65	0.4	0.14	0.2	na	na
19	32	161	f	1	0.9	0.6	0.4	0.02	0.13	na	na
20	21	166	f	0.95	0.7	0.6	0.4	0.13	0.25	–0.5	6.7
21	32	168	m	0.95	0.9	0.58	0.41	0.13	0.23	–0.4	6.5
22	26	176	m	1.1	1	0.67	0.4	0.1	0.17	–0.6	7
23	31	150	f	0.8	0.7	0.51	0.42	0.13	0.2	0.2	5.1
24	29	174	m	1	0.9	0.54	0.4	0.13	0.19	0.2	8.4
25	27	173	m	1	0.9	0.58	0.4	0.1	0.19	–0.8	5.7
26	34	178	m	1.3	1.1	0.71	0.4	0.14	0.2	0.1	5.6
27	26	160	m	0.85	0.8	0.5	0.41	0.23	0.26	0.08	5.7
28	27	170	f	1.25	1	0.65	0.4	0.14	0.19	0.07	9.2
29	32	162	f	1	0.9	0.55	0.4	0.16	0.24	0.6	6
30	23	170	f	1	0.9	0.62	0.4	0.14	0.25	0.3	5.6
Median	28.5	170		1.0	0.9	0.6	0.4	0.12	0.2	0.08	6.3
IQR	5.5	9.5		0.25	0.1	0.1	0.01	0.04	0.04	0.7	2.1
Stats				*p* < 0.001		*p* < 0.001		*p* < 0.001			

m, male; f, female; NW, Normal walking; TW, Target walking; ML, medio-lateral; AP, anterio-posterior; na, not available; IQR, interquartile range; Stats, Statistics; paired wilcoxon test.

### Normal walking

For the Normal walking condition, participants were asked to look straight ahead and sustain a comfortable self-selected walking speed over a 3 min period in the absence of any projected visual targets.

### Statistical analysis

Pooled coherence analyses were performed in order to observe correlation patterns across subjects within each group. Upper and lower 95% confidence limits (CI) are constructed under the assumption of independence and considering the number of segments (L) (Halliday et al., 1995; Amjad et al., 1997):


(3) $$\text{CI}=1-{\left(0.05\right)}^{1/\left(L-1\right)}$$

For pooled data, L denotes the combined number of segments across subjects. Additionally, the χ^2^ extended difference of coherence test (Amjad et al., 1997) was used to explore differences at each frequency between tasks in each cohort. In the control cohort, we found that TW coherence was higher than NW coherence over the frequency range of 8–12 Hz (alpha band) and 21–44 Hz (high-frequency band). Single-subject coherence estimates were averaged from 8–12 to 21–44 Hz for each participant and used to examine intramuscular coherence at the single-subject level in both walking tasks. The high-frequency band from 21 to 44 Hz is of specific interest since it reflects supraspinal information which is transmitted to the motor neuron pools. The mean high-frequency and alpha band coherence estimates were used to investigate the relationships to clinical and biomechanical parameters.

Details on the biomechanical parameters, such as the 3-D CoM trajectory length *C*_*AP–ML–V*_ and the mean Euclidean distance *D*_*AP–ML–V*_, are reported in the Supplementary methods.

The primary outcome was defined as the modulation effect on intramuscular coherence estimates between TW and NW (TW effect) within iSCI individuals in contrast to controls.

The secondary outcome was the correlation between mean intramuscular coherence, clinical parameters, and CoM trajectory length in individuals with iSCI and controls. Finally, the TW effect, the subtraction of NW high-frequency and alpha coherence from TW high-frequency and alpha coherence, was used to correlate with the mean Euclidean distance between TW and NW and TW speed.

Statistical analyses were performed using Matlab (MATLAB R2017b) and SPSS (IBM SPSS Statistics for Macintosh, Version 27.0). Statistical significance was set at α = 0.05. The Kolmogorov–Smirnov test was performed to assess data normality. Two repeated measures ANOVA’s were performed separately for high-frequency and alpha band coherence with the within-subjects variable walking task and the between-subject variable group. For that, we used a variance stabilizing transform of the coherence by applying Fisher’s transform tanh^–1^ to the magnitude coherency (Amjad et al., 1997).

Non-paired Wilcoxon rank-sum tests between individuals with iSCI and controls were performed to investigate differences in CoM trajectory length in each task, and the mean Euclidean distance. Paired Wilcoxon tests were used to investigate differences in CoM trajectory length between NW and TW in individuals with iSCI and controls. The Chi-square test of Independence was used to test for sex differences. Descriptive statistics were also used to compare walking speed, step length and -width, and body height within and between controls and individuals. All reported *p*-values for the demographic and biomechanical data were Bonferroni-corrected per comparison group and reported in the results when significance was not met. Results are shown as the median and interquartile range (IQR). Spearman rank correlations were used to assess the relationship between (1) Mean TW coherence in the (21–44 Hz) high-frequency and alpha (8–12 Hz) band and Max MEP amplitude, (2) Mean TW coherence in the high-frequency and alpha band and mean spinal cord independence measure (SCIM) outdoor score, (3) TW effect (TW – NW high-frequency/alpha coherence) and TW speed, (4) TW effect, mean 3-D Euclidean distance, and (5) mean high-frequency/alpha coherence and 3-D CoM trajectory length. SCIM outdoor score was averaged from the acute to the chronic stage to account for the amount of recovery during rehabilitation.

## Results

### Recruitment

Thirteen participants with subacute or chronic iSCI (ten males; median age 58 years, IQR = 13; one subacute, twelve chronic; see Table 1) and twenty-four controls (14 females; median age 28.5 years, IQR = 5.5; see Table 2) were enrolled in this study. Individuals with iSCI were older than controls (*T* = 400, *n* = 37, *z* = 4.88, *p* < 0.0001, *r* = 0.8) and sex differences were found between the groups [χ^2^(1) = 4.22, *p* = 0.04]. Three iSCI participants who made use of walking aids in everyday walking activity held handrails during both treadmill walking tasks (P01, P10, P15; Table 1). Control participants did not require the use of handrails.

Two individuals with iSCI (not listed in Table 1) were excluded from the analysis due to missing TW data in one case and inconsistent usage of aids during tasks in the other individual. Details on gait parameters are reported in the Supplementary Results.

### Modulation of intramuscular coherence

**FIGURE 2:**
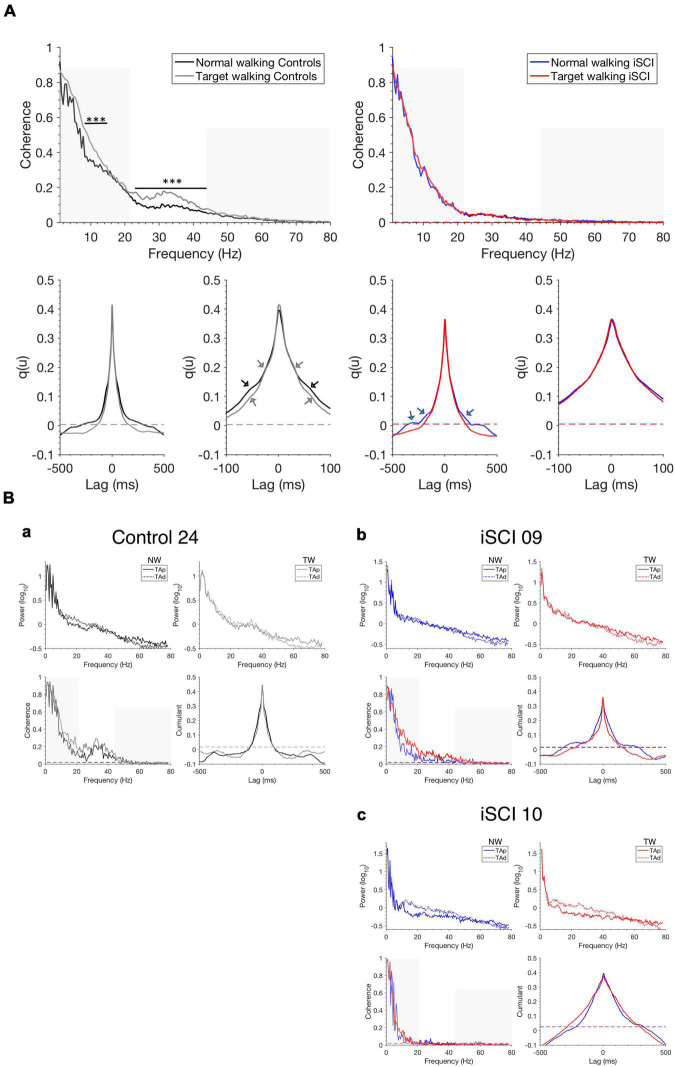
Pooled coherence estimates, single-participant coherence, and cumulant densities during walking. **(A)** Pooled TAp-TAd coherence estimates for controls during NW (black) and TW (gray) (top left) and individuals with iSCI during NW (blue) and TW (red) (top right) and the corresponding cumulants are shown (bottom with expanded lag scales in second and fourth position). The non-shaded area indicates the high-frequency range (21–44 Hz) of interest. The pooled cumulant densities are zoomed to allow for better visualization of secondary features (see arrows). Dashed horizontal lines indicate the upper 95% confidence interval limits. **(B)** Single TAp-TAd coherence estimates, power spectra and cumulant densities for **(a)** a control (Control 24) who shows a median effect of increased mean high-frequency intramuscular coherence (non-shaded area) during TW compared to NW. iSCI 09 **(b)** shows high mean high-frequency coherence (non-shaded area) during TW while iSCI 10 **(c)** shows the lowest mean high-frequency coherence (non-shaded area) during TW. Dashed horizontal lines indicate the upper 95% confidence interval limits. The non-shaded area in the single coherence estimates presents the frequency range of interest 21–44 Hz. Note that the *y*-scale is different for the power spectra in **(Ba–c)**. iSCI, incomplete spinal cord injury; TAp, Tibialis anterior proximal; TAd, Tibialis anterior distal; NW, Normal walking; TW, Target walking. ***P < 0.001.

**FIGURE 3:**
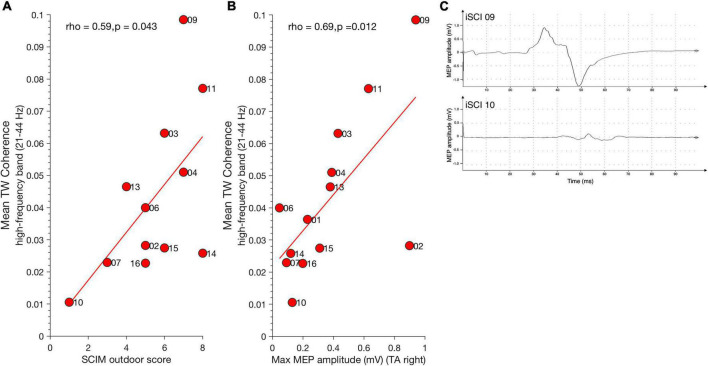
Relationship between high-frequency coherence and clinical parameters. **(A)** Mean SCIM outdoor mobility score positively correlates with high-frequency coherence during TW. **(B)** Maximum MEP amplitude of Tibialis anterior muscle (TA) positively correlates with high-frequency coherence during TW. Dots represent individuals with iSCI and their ID. **(C)** Examples for MEP traces of iSCI 09 (top) who has a high MEP amplitude and high mean high-frequency coherence value and iSCI 10 (bottom) who has low a MEP amplitude and low mean high-frequency coherence value. iSCI, incomplete spinal cord injury; MEP, magnetic evoked potential; SCIM, spinal cord Independence measure; TAp, Tibialis anterior proximal; TAd, Tibialis anterior distal; TA, Tibialis anterior muscle; TW, Target walking.

**FIGURE 4:**
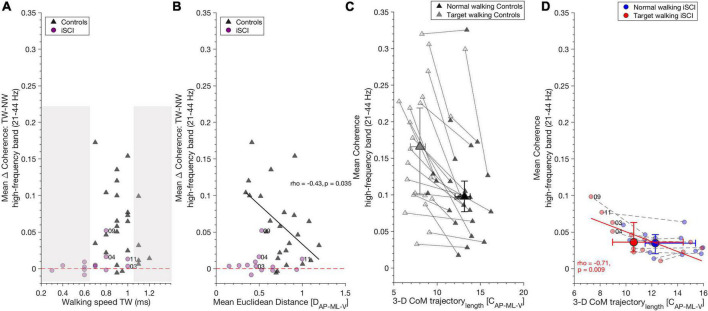
High-frequency coherence is related to gait parameters. **(A)** TW speed did not influence increased high-frequency coherence during TW compared to NW, as shown by the TW effect (Target walking – Normal walking): iSCI (purple dots) and controls (dark gray triangles) increased high-frequency band coherence independent of their walking speed in TW. The non-shaded area depicts iSCI and controls with the same walking speeds. Dashed red line indicates the zero value and distinguishes positive and negative TW effects. **(B)** In controls, increase of high-frequency coherence during TW as shown by TW effect (Target walking – Normal walking) correlated negatively with mean Euclidean distance, whereas in iSCI no correlation was found. Thus, controls who were able to adapt their gait pattern during TW showed decreased TW effect compared to those controls who adapted their gait pattern to a lesser extent. Dashed red line, dots and triangles as in **(A)**. **(C)** A reduction of 3-D CoM trajectory length along with an increase in high-frequency coherence is a typical mechanism found during TW in controls but there is no significant relationship between these parameters. **(D)** However, in individuals with iSCI, 3-D CoM trajectory length during TW negatively correlates with mean TW high-frequency coherence in (red dots). No such relationship was found during NW in individuals with iSCI (blue dots). Gray dotted lines and purple dashed lines connect NW and TW for each control and iSCI, respectively. Dots (individuals with iSCI) and triangles (controls) represent single participants and their ID. Large dots and triangles depict the median and their 95% confidence intervals. iSCI’s ID’s are shown for the skilled or less affected individuals. CoM, center of mass; iSCI, incomplete spinal cord injury; NW, Normal walking; TAp, Tibialis anterior proximal; TAd, Tibialis anterior distal; TW, Target walking. Spearman’s rho and *p*-values are presented where significant; regression lines and 95% confidence limits are omitted for clarity.

Pooled coherence estimates in Figure 2A provide a general summary of the correlation structure in each cohort and task. In the control cohort, we obtained larger coherence magnitudes during TW than NW over the 8–12 Hz and 21–44 Hz range (Figure 2A, left upper panel) (*p* < 0.05), whereas in the iSCI cohort no differences were obtained between TW and NW (Figure 2A, right upper panel) (*p* > 0.05). Furthermore, lower intramuscular coherence magnitudes were obtained in the iSCI cohort compared to the control cohort. The cumulant density structure (Figure 2A, lower panel, second from left) in controls displayed secondary rhythmic peaks at –58/+62 ms for NW and TW and ±30 ms for TW, consistent with the corresponding periodicities of 17.2/16.1 and 33.3 Hz, respectively, found in the coherence plot (Figure 2A, left upper panel) and were accompanied by the enhanced central peak at time lag 0 ms (Halliday et al., 1995). In the iSCI cohort, the cumulant density structure for NW showed secondary peaks at ± 190 ms and –310 ms, corresponding to 5.3 and 3.2 Hz, respectively, accompanied by a central peak at time lag 0 ms (Figure 2A, lower panel, third from left). The TW effect (calculated by subtracting mean NW coherence from mean TW coherence in the high-frequency band in single participants) was significantly higher in healthy controls compared to individuals with iSCI (*T* = 44, *n* = 37, *z* = –3.56, *p* < 0.001, *r* = –0.59). To illustrate this, examples of the analysis obtained from a control and two individuals with iSCI are given below (Figure 2B) and how variation in coherence measures across the iSCI group relates to clinical assessment is explored in the next sections (Figures 3, 4). In a single control (Control 24, Figure 2Ba), who showed a median TW effect, we found an enhanced modulation of intramuscular coherence in the 21–44 Hz range. This modulation was also reflected in the respective EMG power spectra and the cumulant had a clear sharp peak at time lag 0 ms. Such enhanced modulation of intramuscular coherence in the frequency band 21–44 Hz was lacking or reduced to various degrees in individual individuals with iSCI (Figures 2Bb,c): While iSCI 09 (Figure 2Bb) showed increased intramuscular coherence during TW compared to NW with a pointed peak in the cumulant density plot at time lag 0 ms, iSCI 10 (Figure 2Bc) showed little intramuscular coherence for TW and NW and the cumulant density plot was broader compared to that of iSCI 09 and Control 24. The repeated measures ANOVA with a Greenhouse–Geisser correction determined that TW high-frequency coherence was significantly higher than NW high-frequency coherence [*F*(1.0,35.0) = 25.705, *p* < 0.001]. Furthermore, there was a statistically significant interaction between TW and NW high-frequency coherence and group [*F*(1.0,35.0) = 13.042, *p* < 0.001]. The repeated measures ANOVA with a Greenhouse–Geisser correction determined that TW alpha band coherence was significantly higher than NW alpha band coherence [*F*(1.0,35.0) = 17.147, *p* < 0.001]. However, there was no statistically significant interaction between TW and NW alpha band coherence and group [*F*(1.0,35.0) = 3.266, *p* = 0.079]. Thus, the change observed between TW and NW alpha band coherence does not depend on the type of group and further analyses on the alpha band coherence should be interpreted cautiously.

### Relationship between coherence and clinical/neurophysiological parameters in individuals with incomplete spinal cord injury

Spinal cord independence measure outdoor mobility score positively correlated with mean TW high-frequency coherence (21–44 Hz) [spearman’s ρ(11) = 0.59, *p* = 0.043] (Figure 3A) but not with mean NW high-frequency coherence [spearman’s ρ(11) = 0.56, *p* = 0.059]. Thus, individuals with high mean SCIM outdoor scores exhibited greater high-frequency coherence in the TW condition. Significant mean high-frequency coherence estimates obtained during TW correlated positively with maximum MEP amplitude of the right Tibialis anterior muscle [spearman’s ρ(11) = 0.69, *p* = 0.012] (Figure 3B). This is in line with observations previously demonstrating a relationship between MEP amplitude, Tibialis anterior coherence, and ankle dorsiflexion during the swing phase of walking in individuals with iSCI (Barthélemy et al., 2010). There was no relationship between TW high-frequency coherence modulation and MEP latency [spearman’s ρ (11) = –0.36, *p* = 0.224]. Figure 3C shows examples of one iSCI individual with a relatively high MEP amplitude and high-frequency coherence (iSCI 09, top) and another iSCI individual with low MEP amplitude and high-frequency coherence (iSCI 10, bottom). High-frequency coherence obtained in NW did not correlate with MEP amplitude [spearman’s ρ(11) = 0.55, *p* = 0.053] and latency [spearman’s ρ(11) = –0.26, *p* = 0.373].

Motor-evoked potentials amplitude correlated positively with mean TW alpha band coherence (8–12 Hz) [ρ(11) = 0.74, *p* = 0.006] but not with NW alpha band coherence [ρ(11) = 0.07, *p* = 0.835]. No significant correlation was found between mean alpha band coherence and MEP latency for TW [ρ(11) = –0.33, *p* = 0.271] and NW [ρ(11) = 0.03, *p* = 0.935]. Furthermore, no significant correlation was found between mean alpha band coherence and SCIM for TW [ρ(11) = 0.24, *p* = 0.451] and NW [ρ(11) = 0.18, *p* = 0.582].

To check whether TW effects are solely obtained for the high-frequency coherence (beta and gamma band, 21–44 Hz), we calculated the mean coherence in the low beta frequency range (13–21 Hz) for TW and NW. MEP amplitude was not correlated with mean low beta frequency coherence during TW [ρ(11) = 0.44, *p* = 0.135], nor during NW [ρ(11) = 0.46, *p* = 0.119].

### High-frequency band coherence is related to gait parameters during Target walking

Target walking effect was calculated by subtracting mean NW coherence from mean TW coherence in the high-frequency band and alpha band (the resulting value describes the increase of coherence during TW).

Target walking speed in the control group or in iSCI did not influence the increase of high-frequency coherence seen during TW in controls [spearman’s ρ(22) = –0.24, *p* = 0.260] or iSCI [spearman’s ρ(11) = 0.46, *p* = 0.11] (Figure 4A) nor the increase of alpha band coherence during NW in controls [spearman’s ρ(22) = –0.06, *p* = 0.789] or iSCI [spearman’s ρ(11) = 0.13, *p* = 0.66]. As the non-shaded area indicates in Figure 4A, iSCI and controls with the same TW speed exhibited different TW effects. The mean Euclidean distance was negatively correlated with the TW effect of high-frequency coherence in controls [spearman’s ρ(22) = –0.43, *p* = 0.035] (Figure 4B). Thus, controls who reduced their CoM movement in the TW task and obtained higher mean Euclidean distances between TW and NW exhibited lower TW effects (smaller coherence difference between TW and NW in the high-frequency band). Mean Euclidean distance did not affect TW effect in individuals with iSCI [spearman’s ρ(11) = –0.03, *p* = 0.92]. The TW effect of alpha band coherence was not significantly correlated with the mean Euclidean distance in either controls [spearman’s ρ(22) = –0.11, *p* = 0.96] or iSCI [spearman’s ρ(11) = 0.13, *p* = 0.682].

A significant reduction of TW 3-D CoM trajectory length was observed during TW compared to NW in controls. However, no relationship was found with high-frequency coherence for either task [NW: spearman’s ρ(22) = –0.09, *p* = 0.673; TW: spearman’s rho(22) = 0.12, *p* = 0.586] (Figure 4C) or alpha band coherence [NW: spearman’s ρ(22) = –0.19, *p* = 0.371; TW: spearman’s rho(22) = –0.13, *p* = 0.536]. In individuals with iSCI, 3-D CoM trajectory length on average did not differ between the tasks. However, TW 3-D CoM trajectory length negatively correlated with mean high-frequency coherence during TW [spearman’s ρ(11) = –0.71, *p* = 0.009] (Figure 4D). Thus, the individuals with iSCI who reduced their CoM trajectory length during TW generated greater high-frequency coherence. No significant relationship was found between NW high-frequency coherence and NW 3-D CoM trajectory length in individuals with iSCI [spearman’s ρ(11) = –0.43, *p* = 0.15] (Figure 4D). The alpha band coherence during TW correlated negatively with TW 3-D CoM trajectory length [spearman’s ρ(11) = –0.62, *p* = 0.029] but not during NW with NW 3-D CoM trajectory length [spearman’s ρ(11) = –0.25, *p* = 0.404] in individuals with iSCI.

Accuracy in ML-direction differed (T = 209.5, *n* = 24, *z* = 3.41, *p* = 0.0006, *r* = 0.7) between individuals with iSCI and controls, whereas no difference was found in AP-direction (*T* = 151, *n* = 24, *z* = 0, *p* > 0.05, *r* = 0).

Mean high-frequency band coherence did not correlate with task performance (accuracy) in controls [AP: spearman’s ρ(22) = –0.22, *p* = 0.499, ML: spearman’s ρ(22) = 0.45, *p* = 0.14] or individuals with iSCI [AP: spearman’s ρ(11) = 0.54, *p* = 0.075, ML: spearman’s ρ(11) = –0.39, *p* = 0.210] nor did the alpha band in controls [AP: spearman’s ρ(22) = –0.06, *p* = 0.85, ML: spearman’s ρ(22) = –0.24, *p* = 0.46] or individuals with iSCI [AP: spearman’s ρ(11) = 0.27, *p* = 0.40, ML: spearman’s ρ(11) = –0.36, *p* = 0.256].

## Discussion

This study demonstrates that the magnitude of high-frequency (21–44 Hz) intramuscular coherence over the gait cycle is enhanced during TW and systematically related to adjustments affecting balance control in space (i.e., Euclidean distance between TW and NW) in a control group. However, in the iSCI cohort, high-frequency modulation was severely diminished, indicating impaired task adaptability. Interestingly, in iSCI individuals who regained reasonable skilled walking abilities, high-frequency coherence was preserved reflecting neural adaptability of gait potentiated during TW. This is the first experimental account of a correlation of high-frequency intramuscular coherence with biomechanical proxies for volitional gait adaptation and its systematic correlation with neurophysiological (MEP) and functional scores (self-reported walking performance in SCIM) in iSCI.

### Intramuscular coherence during Target walking reflects visuomotor integration

In this study, TW is a proxy for complex outdoor walking where participants need to voluntarily modify their step length and width to accommodate obstacles and adjust the step cycle to the terrain (Dietz, 1992; Drew and Marigold, 2015).

Corticomuscular coherence in high-frequency bands, namely the beta and gamma spectra, is associated with supra-spinal input (Conway et al., 1995; Salenius et al., 1997; Brown et al., 1998; Petersen et al., 2012) as it is modulated by demanding tasks (Brown et al., 1998) requiring attention (Johnson et al., 2011) and precision (Kristeva-Feige et al., 2002). In support of this notion, increased corticomuscular coherence at 15–35 Hz has been observed after visuomotor skill training of the ankle dorsiflexor (Perez et al., 2006). In individuals with iSCI performing regular treadmill walking, previous studies have shown reduced or absent intramuscular TAp-TAd coherence in the high-frequency band, which was attributed to compromised descending motor input (Hansen et al., 2005; Barthélemy et al., 2010). In addition, in this study, the high-frequency coherence modulation as observed in the control subjects was not uniformly expressed in individuals with iSCI. In a majority of iSCI individuals, modulation was lacking during TW (for instance, iSCI 10), while it could be observed in some individuals (for instance, iSCI 09). Interestingly, the individual differences appear to relate to the capability of individuals to adjust their gait to challenging Targeted walking tasks. This finding is novel and expands on previous studies demonstrating the reduction of high-frequency coherence during NW in individuals with iSCI.

The results of the coherence analysis in the iSCI cohort are supported by cumulant estimates, which reveal variations in the size and width of the central peak. Both the central peak amplitude and width in the cumulant are indicative of time domain features that reveal variations in the degree and frequency content of descending inputs contributing to synchronization within the recorded Tibialis anterior EMG (Hansen et al., 2005).

In contrast to the high-frequency observations, the alpha band coherence change from NW to TW was not different between the groups. One reason for this may be that spinal interneurons that drive the spinal motoneurons remain functional in individuals with iSCI (Hansen et al., 2005).

### Intramuscular coherence and its relationship to gait adaptability mechanisms in controls and individuals with incomplete spinal cord injury

Effective capacity to adapt gait to external demands can be assessed with the SCIM outdoor mobility score (van Hedel and Dietz, 2009). We found a positive correlation between mean SCIM outdoor score and high-frequency band coherence during TW in individuals with iSCI, indicating that coherence corresponds to self-experienced walking abilities. This is supported by previous results, showing that the ability to reduce the TW 3-D CoM trajectory length correlated with the mean SCIM outdoor mobility score (Mohammadzada et al., 2022). Walking speed is commonly used to quantify walking capacity in individuals with iSCI (van Hedel et al., 2007; van Silfhout et al., 2017) and was lower in individuals with iSCI than in controls. However, in this study, TW speed was unrelated to the increase of intramuscular high-frequency coherence during TW. This is an expected result as walking speed is generally represented by intramuscular coherence in low-frequency bands that capture features related to gait rhythm and the overall EMG burst envelope, while high-frequency features are generally considered to provide insight into common EMG activity features that occur within EMG bursts. Accordingly, the difference in TW speed between both groups cannot explain the difference in high-frequency coherence found for individuals with iSCI and controls as demonstrated by iSCI and controls walking at similar TW speeds (see Figure 4A).

Adjustments of 3-D CoM movement to TW demand were assessed by estimating the mean Euclidean distance between TW and NW. We found that those controls showing high mean Euclidean distance by reducing 3-D CoM movement during TW exhibited smaller increases in high-frequency coherence, thus, smaller relative TW effects on coherence (see Figure 4B). As such, a reduction of CoM movement was also found for individuals with iSCI despite their lack of coherence increase; this may point to a strategy to adapt to the TW demands, which may not necessarily rely on an increment of common rhythmic central drive. It may nevertheless involve preserved capacity for supra-spinal control given iSCI’s capability to reduce their CoM trajectory length during TW correlated negatively with TW high-frequency coherence (see TW in individuals with iSCI, Figure 4D). This suggests that TW provokes a higher demand on the common central drive through intact fibers of descending tracts to the muscles coupled to 3-D CoM trajectory length in some (less affected) individuals with iSCI (i.e., 03, 04, 09, 11). However, this may also indicate that the requirements necessary for the performance of a challenging walking task are coupled to anatomical and/or biomechanical constraints in individuals with iSCI. Thus, individuals with iSCI may be functionally restricted in their ability to adapt their walking pattern during TW due to impaired intralimb coordination, muscle weakness, proprioception (Awai and Curt, 2014), and level of injury (Mohammadzada et al., 2022).

Performance accuracy did not correlate with intramuscular TAp-TAd coherence. We assume that the mere demand of the task was enough to increase high-frequency and alpha band coherence, irrespective of how well the participants hit the target. Furthermore, intramuscular coherence when measured in Tibialis anterior alone may not be sensitive enough to detect minimal errors in ML direction and errors exaggerated in AP direction due to the nature of the task. Studies on the behavior of other muscle groups contributing to successful target hits, particularly in the ML direction are warranted.

### The interplay of intramuscular coherence and motor-evoked potentials

In individuals with iSCI, high-frequency and alpha band coherence during TW positively correlated with MEP amplitude, which suggests that individuals with preserved corticospinal integrity adapt better to the challenging walking task.

It has been previously demonstrated that following locomotor training, individuals with iSCI with moderate muscle strength showed increases in maximum MEP size that correlated positively with 24-40 Hz coherence (Norton and Gorassini, 2006). The authors proposed that the increase in high-frequency (24–40 Hz) coherence may be mediated via spared corticospinal tract function in these individuals. Furthermore, as MEPs and intramuscular coherence in Tibialis anterior during early swing have been shown to be correlated with the degree of foot drop in single individuals with iSCI (Barthélemy et al., 2010), it is likely that in TW tasks, where precision in foot placement is a requirement, coherence modulation throughout swing is enhanced. The correlation to MEP amplitude was also present for the alpha band coherence during TW indicating that TW leads to an overall excitability change in neural systems that contribute to the generation of gait in iSCIs. However, in contrast to the high-frequency coherence, the difference between NW and TW alpha band coherence was not significant between the groups, suggesting that the former is a manifestation of mechanisms related to the TW task.

In our cohort, individuals 03, 04, 09, and 11 showed increased high-frequency band coherence during TW. We interpret this as an indication not only that a corticospinal innervation remains active in these individuals but that the innervation is capable of transmitting synchronizing inputs over the range of frequencies represented in the coherence results. In these individuals, TW may have recruited residual corticospinal drive, whilst in those individuals who failed to show enhanced high-frequency coherence together with small or delayed MEPs any residual corticospinal drive may be incapable of sustaining high-frequency synchronization within spinal motor pools. However, given the complex interplay of the corticospinal tract, the extrapyramidal motor system, reticulospinal, and ascending sensory tracts, their contribution to coherence needs to be further investigated. Seven out of 13 individuals with iSCI (01, 02, 04, 10, 14, 15, 16) had sensory scores lower than 100 points (max is 112), whereas the others had higher scores. Of those individuals with low sensory scores, the majority (5 out of 7; 02, 10, 14, 15, 16) had mean high-frequency TW coherence magnitudes lower than 0.03. This points toward an interrelation between sensory impairment and low levels of high-frequency coherence. Previous studies support this notion. For instance, modulation of afferent input via arm cooling has been shown to reduce beta-band corticomuscular coherence in hand- and arm muscles in some controls (Riddle and Baker, 2005). Similarly, digital nerve anesthesia of the hand muscle led to a decrease of beta-band intermuscular coherence in controls (Fisher et al., 2002). The lack of the afferent input also leads to a reduction of intermuscular coherence in the beta range (15–30 Hz) shown in studies investigating a deafferented patient (Kilner et al., 2004; Schmied et al., 2014).

### Limitations

Incomplete spinal cord injury characteristics were different in terms of etiology, level of injury, and central and peripheral neurological pathologies. In addition, controls were neither sex-, age-, nor walking speed-matched.

Age may have affected the results, although its effects on intramuscular beta band coherence are ambiguous (Semmler et al., 2003; Jaiser et al., 2016; Spedden et al., 2018; Watanabe et al., 2018) and we found significant sex differences between the cohorts. Moreover, our results suggest that the decrease in intramuscular coherence during TW and NW is due to the individual’s neurological impairment since similar results have been obtained in previous studies during NW (Hansen et al., 2005; Barthélemy et al., 2010).

The TW task was not adapted to individual step width and length, which may be comparable to challenges faced when walking outdoors in a natural environment and may similarly affect individuals with iSCI and controls. We are confident that the measurements and analysis conducted were not affected by cross-talk between the muscle pairs, which, if present, would be characterized by significant high-amplitude broad-band coherence and a narrow central peak in the cumulant density (Hansen et al., 2001; Halliday et al., 2003). CoM calculation was not based on a full-body marker set because reliable placement of the torso markers was prevented by the safety harness necessary for this iSCI population. Nevertheless, the approximated CoM model is well understood in its limitations (Gard et al., 2004; Süptitz et al., 2013; Havens et al., 2018).

Although the Tibialis anterior muscle is mainly active during the swing phase of gait, we analyzed intramuscular TAp-TAd coherence for the entire gait cycle. We were interested in the correlation pattern across the entire gait cycle since gait abnormalities in iSCI are not restricted to specific gait phases. However, extending the study to investigate intermuscular coherence between additional lower limb muscles during TW is warranted in order to determine which specific phases of the gait cycle increased high-frequency coherence contributes most. Furthermore, we aimed at investigating the dynamic relationship with the CoM parameters which considers the entire gait cycle as well. We further focused on the intramuscular coherence of the Tibialis anterior since this ankle dorsiflexor muscle is proposed to receive neural drive from supra-spinal systems as shown in controls (Schubert et al., 1997; Halliday et al., 2003; Spedden et al., 2019b) as well as pathological walking (Hansen et al., 2005; Nielsen et al., 2008). Spasticity, which may alter the walking pattern and -capacity in individuals with iSCI (Krawetz and Nance, 1996), was not assessed. The comparison to EEG-EMG coherence literature is limited, as frequency bands were determined in a data-driven approach and not through predefined ranges commonly used in EEG. Future studies are required to investigate the test-retest reliability of intramuscular coherence during TW, as one previous study pointed out limits of agreement and reliability under specific conditions during NW (van Asseldonk et al., 2014).

## Conclusion

To our knowledge, this is the first study to demonstrate an interplay between intramuscular coherence and biomechanical features of gait, here quantified by CoM control. Individuals with iSCI generated less coherence in high-frequency bands during TW than controls whereas no difference was found for the alpha band. Furthermore, the extent of reduced modulated high-frequency coherence was related to MEP pathologies, biomechanical proxies for the gait disturbance, and self-reported walking performance (SCIM).

Thus, intramuscular coherence during TW could be used to quantify preserved supra-spinal control in individuals with iSCI since it quantifies subtle gait disturbances. Intramuscular coherence may provide a complementary insight into the recovery of gait function beyond gross motor scores (such as muscle strength and walking distances) where a targeted advancement of fine motor control may benefit gait rehabilitation strategies.

## Data availability statement

The original contributions presented in this study are included in the article/Supplementary material, further inquiries can be directed to the corresponding author.

## Ethics statement

The studies involving human participants were reviewed and approved by Zurich Cantonal Ethics Committee. The patients/participants provided their written informed consent to participate in this study.

## Author contributions

FZ-M, CE, and MS designed the study. FZ-M, MS, CZ, CE, BC, DH, and AC assisted in data interpretation, manuscript preparation, and manuscript revision. FZ-M recruited participants and conducted measurements with assistance of CE. CZ and MS did the clinical assessment of individuals with iSCI. FZ-M performed the data analysis and wrote the manuscript. All authors read and approved the final version of the manuscript.

## Footnotes

1
https://www.neurospec.org/
