# Supplementary material for: Intramuscular coherence during challenging walking in incomplete spinal cord injury: Reduced high-frequency coherence reflects impaired supra-spinal control
Source: Front Hum Neurosci. 2022 Aug 3;16:927704. doi: 10.3389/fnhum.2022.927704 (PMC9387543; doi:10.3389/fnhum.2022.927704)
Supplement: Supplementary file 1 [file Data_Sheet_1.DOCX]

Supplementary Material

# Supplementary Methods

## *Data analysis*

The biomechanics of the walking tasks were investigated using the CoM. We previously described the CoM parameters thoroughly during TW and NW in the same iSCI cohort and twelve controls (Mohammadzada et al., 2022). For the investigation of systematic relationships to the intramuscular coherence estimates, two CoM parameters were chosen to reflect the body movement during walking which will be explained in detailed in the following. CoM trajectories in AP, ML, and vertical (V) directions were time-normalized to 100% gait cycle from HS to HS, using linear normalization with an anchor at the end of the stance phase. The anchor was used to prevent warping of the trajectory through changes in relative stance and swing durations. Trajectories were then averaged for all recorded steps, centered by subtracting their mean and normalized by body height. For each participant and walking condition, the total length of the 3-D CoM trajectory length $C_{AP-ML-V}$ was calculated as the summed Euclidean distance between samples in time (*i*: sample number, *n*: number of total samples). $C_{AP-ML-V}$ is defined as

| $\boldsymbol{C}_{\boldsymbol{AP-ML-V}}\boldsymbol{=}\sum_{\boldsymbol{i=1}}^{\boldsymbol{n-1}} \sqrt{\boldsymbol{(}{\boldsymbol{AP}_{\boldsymbol{i+1}}\boldsymbol{-}\boldsymbol{AP}_{\boldsymbol{i}}\boldsymbol{)}}^{\boldsymbol{2}}\boldsymbol{+(}{\boldsymbol{ML}_{\boldsymbol{i+1}}\boldsymbol{-}\boldsymbol{ML}_{\boldsymbol{i}}\boldsymbol{)}}^{\boldsymbol{2}}\boldsymbol{+(}{\boldsymbol{V}_{\boldsymbol{i+1}}\boldsymbol{-}\boldsymbol{V}_{\boldsymbol{i}}\boldsymbol{)}}^{\boldsymbol{2}}}$ | (4) |
| --- | --- |

Differences in CoM shape pattern between Target (TW) and Normal walking (NW) were calculated as the mean Euclidean distance $D_{AP-ML-V}$ between the conditions at each point in time (*i*) for each participant

| $\boldsymbol{D}_{\boldsymbol{AP-ML-V}}\boldsymbol{=}\frac{\sum_{\boldsymbol{i=1}}^{\boldsymbol{n}} \sqrt{\boldsymbol{(}{\boldsymbol{AP}_{\boldsymbol{i;TW}}\boldsymbol{-}\boldsymbol{AP}_{\boldsymbol{i;NW}}\boldsymbol{)}}^{\boldsymbol{2}}\boldsymbol{+(}{\boldsymbol{ML}_{\boldsymbol{i;TW}}\boldsymbol{-}\boldsymbol{ML}_{\boldsymbol{i;NW}}\boldsymbol{)}}^{\boldsymbol{2}}\boldsymbol{+(}{\boldsymbol{V}_{\boldsymbol{i;TW}}\boldsymbol{-}\boldsymbol{V}_{\boldsymbol{i;NW}}\boldsymbol{)}}^{\boldsymbol{2}}}}{\boldsymbol{n}}$ | (5) |
| --- | --- |

A large mean distance indicates a large change in CoM shape pattern during TW compared to NW, and thus an increased adaptation to TW, whereas a small mean distance indicates similar CoM shape patterns between TW and NW, thus a smaller adaptation to TW.

# Supplementary Results

***Gait parameters***

In individuals with iSCI, the median self-selected speed for NW was 0.8 m/s (IQR=0.2) and not significantly different from median TW speed 0.7 m/s (IQR=0.2) (T=34.5, n=13, z=2.35, p=0.02, r=0.65; adjusted p-value p<0.012) (Supplementary Table1).

The median self-selected walking speed across all controls for NW was 1 m/s (IQR=0.25) and significantly higher than their median TW speed of 0.9 m/s (IQR=0.1) (T=210, n=24, z=3.98, p<0.0001, r=0.81) (Supplementary Table 2). Walking speeds were lower in individuals with iSCI than controls for NW (T=126.5, n=37, z=-3.86, p=0.0001, r=-0.63) and TW (T=136.5, n=37, z=-3.56, p=0.0003, r=-0.59). During the TW task, target presentation sequences largely determine step cycle metrics and consequently step length was seen to be significantly greater during NW compared to TW in iSCI (T=82.5, n=13, z=2.59, p=0.01, r=0.72) and controls (T=300, n=24, z=4.29, p<0.0001, r=0.88), whereas step width was larger during TW when compared to NW in iSCI (T=2, n=13, z=-3.05, p=0.002, r=-0.85) and controls (T=0, n=24, z=-4.3, p<0.0001, r=-0.88). Step length was smaller in individuals with iSCI than controls during NW (T=150.5, n=37, z=-3.08, p=0.002, r=0.51) but not during TW (T=223.5, n=37, z=-0.8, p=0.425, r=-0.13). Step width was smaller in individuals with iSCI than in controls during TW (T=151.5, n=37, z=-3.11, p=0.002, r=-0.51) but not during NW (T=292, n=37, z=1.44, p=0.15, r=0.24).

In controls, the length of 3-D CoM trajectory during TW was smaller than during NW (T=300, n=24, z=4.29, p<0.0001, r=0.88), whereas in iSCI, no difference between NW and TW 3-D CoM trajectory length was found (T=74, n=13, z=1.99, p=0.046, r=0.55; adjusted p-value p<0.012). The 3-D CoM trajectory length in individuals with iSCI was greater compared to controls for TW (T=353, n=37, z=3.37, p=0.001, r=-0.55) but equal during NW (T=254, n=37, z=0.22, p=0.836, r=-0.036). The mean Euclidean distance between TW and NW in controls was greater than in iSCI (T=172, n=37, z=-2.39, p=0.018, r=-0.39).

In individuals with iSCI, the median number of gait cycles used for coherence analysis for NW was 152 (IQR=27.5) and for TW 159 (IQR=40). In controls, the median number of gait cycles for NW was 161 (IQR=22.5) and for TW 140 (IQR=17.5). Examples of lower limb kinematics and EMG modulation for NW are shown in the Supplementary Results (Supplementary Figure 1).

## Supplementary Figures

**Supplementary Figure 1: Modulation of lower limb kinematics and Tibialis anterior (TA) EMG during Normal walking.** Ankle, knee, and hip joint positions are shown together with EMG activity from the Tibialis anterior proximal (TAp) and distal (TAd) for one control (Control 24) and one individual with iSCI (iSCI 10). iSCI: incomplete spinal cord injury; TAp: Tibialis anterior proximal; TAd: Tibialis anterior distal.

***References***

MOHAMMADZADA, F., ZIPSER, C. M., EASTHOPE, C. A., HALLIDAY, D. M., CONWAY, B. A., CURT, A. & SCHUBERT, M. 2022. Mind your step: Target walking task reveals gait disturbance in individuals with incomplete spinal cord injury. *J Neuroeng Rehabil,* 19**,** 36.
